# Supplementary material for: Genome-Wide Identification and Functional Analysis of the GUX Gene Family in Eucalyptus grandis
Source: Int J Mol Sci. 2024 Jul 27;25(15):8199. doi: 10.3390/ijms25158199 (PMC11311485; doi:10.3390/ijms25158199)
Supplement: Supplementary file 1 [file ijms-25-08199-s001.zip › Table S1 S2.pdf]

Table S1 Mapping table of gene name and gene ID

Scientific name	Accession number	Phylogenetic tree name
Selaginella moellendorffii	EFJ12427.1	Selaginella_moellendorffii_GUX1
	EFJ38373.1	Selaginella_moellendorffii_GUX2
	EFJ06418.1	Selaginella_moellendorffii_GUX3
	EFJ18775.1	Selaginella_moellendorffii_GUX4
	EFJ38142.1	Selaginella_moellendorffii_GUX5
	EFJ38143.1	Selaginella_moellendorffii_GUX6
	EFJ11471.1	Selaginella_moellendorffii_GUX7
	EFJ08771.1	Selaginella_moellendorffii_GUX8
	EFJ27644.1	Selaginella_moellendorffii_GUX9
	EFJ34085.1	Selaginella_moellendorffii_GUX10
	EFJ37303.1	Selaginella_moellendorffii_GUX11
	EFJ29334.1	Selaginella_moellendorffii_GUX12
	EFJ29333.1	Selaginella_moellendorffii_GUX13
	EFJ14850.1	Selaginella_moellendorffii_GUX14
Physcomitrium patens	XP_024370263.1	Physcomitrium_patens_GUX1
	XP_024392627.1	Physcomitrium_patens_GUX2
	XP_024392626.1	Physcomitrium_patens_GUX3
	XP_024392628.1	Physcomitrium_patens_GUX4
	XP_024397736.1	Physcomitrium_patens_GUX5
	XP_024392623.1	Physcomitrium_patens_GUX6
	XP_024392624.1	Physcomitrium_patens_GUX7
	XP_024369370.1	Physcomitrium_patens_GUX8
	XP_024368368.1	Physcomitrium_patens_GUX9
	XP_024366927.1	Physcomitrium_patens_GUX10
	XP_024369371.1	Physcomitrium_patens_GUX11
	XP_024392932.1	Physcomitrium_patens_GUX12
	XP_024384256.1	Physcomitrium_patens_GUX13
	XP_024369368.1	Physcomitrium_patens_GUX14
	XP_024389625.1	Physcomitrium_patens_GUX15
	XP_024369369.1	Physcomitrium_patens_GUX16
	XP_024369707.1	Physcomitrium_patens_GUX17
	XP_024369708.1	Physcomitrium_patens_GUX18
Arabidopsis thaliaba	AT1G08990.1	Arabidopsis_thaliaba_GUX5
	AT1G54940.1	Arabidopsis_thaliaba_GUX3
	AT1G77130.1	Arabidopsis_thaliaba_GUX4
	AT3G18660.2	Arabidopsis_thaliaba_GUX1
	AT4G33330.2	Arabidopsis_thaliaba_GUX2

Populus alba	evm.model.Poalb10G012790.1	Populus_alba_GUX1
	evm.model.Poalb13G007470.1	Populus_alba_GUX2
	evm.model.Poalb05G004620.1	Populus_alba_GUX3
	evm.model.Poalb02G012620.1	Populus_alba_GUX4
	evm.model.Poalb14G002590.1	Populus_alba_GUX5
	evm.model.Poalb03G006370.1	Populus_alba_GUX6
	evm.model.Poalb01G014250.1	Populus_alba_GUX7
	evm.model.Poalb05G000180.1	Populus_alba_GUX8
	evm.model.Poalb08G008340.1	Populus_alba_GUX9
	evm.model.Poalb07G011530.1	Populus_alba_GUX10
	evm.model.Poalb13G006070.1	Populus_alba_GUX11
	evm.model.Poalb05G000630.1	Populus_alba_GUX12
	evm.model.Poalb13G006060.1	Populus_alba_GUX13
	evm.model.Poalb05G015230.1	Populus_alba_GUX14
	evm.model.Poalb14G009520.1	Populus_alba_GUX15
	evm.model.Poalb13G009860.1	Populus_alba_GUX16
	evm.model.Poalb10G003340.1	Populus_alba_GUX17
	evm.model.Poalb05G002270.1	Populus_alba_GUX18
	evm.model.Poalb08G016390.1	Populus_alba_GUX19
	evm.model.Poalb02G006990.1	Populus_alba_GUX20

Table S2 The name, sequence, and functionality of the promoter regions

Element name	Element sequence	Element function
3-AF1 binding site	TAAGAGAGGAA	light responsiveness
AAAC-motif	CAATCAAAACCT	
ACE	GACACGTATG; GCGACGTACC	
AE-box	AGAAACAA; AGAAACTT	
ATC-motif	AGTAATCT	
ATCT-motif	AATCTAATCC	
Box 4	ATTAAT	
Box II	ACACGTTGT	
chs-CMA1a	TTACTTAA	
GA-motif	ATAGATAA	
Gap-box	CAAATGAA(A/G)A	
GATA-motif	GATAGGA; AAGGA	
G-box	TAAGG; GATAGGG; AAGATAAGATT tgACACGTGGCA; ACACGTGGC; CACGTG; GCCACGTGGA; TACGTG; CACGTC; TAACACGTAG; CACGAC; ACACGTGT; CACGTT	
GT1-motif	GGTTAA; GGTTAAT	
I-box	AGATAAGG; cCATATCCAAT; AAGATAAGGCT; cGATAAGGCG; ccttatcct; TGATAATGT; atGATAAGGTC	
LAMP-element	CTTTATCA	
L-box	ATCCCACCTAC	
Sp1	GGGCGG	
TCCC-motif	TCTCCCT	
TCT-motif	TCTTAC	
CAT-box	GCCACT	meristem expression
TGACG-motif	TGACG	MeJA-responsiveness
CGTCA-motif	CGTCA	
LTR	CCGAAA	low-temperature responsiveness
Unnamed_1	GGATTTTACAGT	phytochrome down-regulation expression
O2-site	GATGACATGG; GTTGACGTGA; GATGA(C/T)(A/G)TG(A/G); GATGATGTGG	zein metabolism regulation
ABRE	GCAACGTGTC; CACGTG; ACGTG; GACACGTGGC; GCCGCGTGGC; AACCCGG; CGCACGTGTC; TACGGTC	abscisic acid responsiveness
circadian	CAAAGATATC	circadian control
AuxRR-core	GGTCCAT	auxin responsiveness

TC-rich repeats	GTTTTCTTAC；ATTCTCTAAC	defense and stress responsiveness
P-box	CCTTTTG	gibberellin-responsiveness
ARE	AAACCA	anaerobic induction
TCA-element	CCATCTTTTT；TCAGAAGAGG	salicylic acid responsiveness
SARE	TTCGACCATCTT	
GC-motif	CCCCCG	anoxic specific inducibility
GCN4_motif	TGAGTCA	endosperm expression
WUN-motif	AAATTTCCT	wound-responsive element
MSA-like	TCCAACGGT	cell cycle regulation
AT-rich sequence	TAAAATACT	element for maximal elicitor-mediated activation (2copies)
MRE	AACCTAA	MYB binding site involved in light responsiveness
MBS	CAACTG	MYB binding site involved in drought-inducibility
MBSI	TTTTTACGGTTA；aaaAaaC(G/C)GTTA	MYB binding site involved in flavonoid biosynthetic genes regulation
DRE	TACCGACAT	cis-acting element involved in dehydration, low-temp, salt stresses
